# Mycoplasmoses of ruminants in France: recent data from the national surveillance network

**DOI:** 10.1186/1746-6148-6-32

**Published:** 2010-06-07

**Authors:** Myriam Chazel, Florence Tardy, Dominique Le Grand, Didier Calavas, François Poumarat

**Affiliations:** 1 UMR Ruminant mycoplasmoses AFSSA-Lyon, 31 avenue Tony Garnier 69364 Lyon cedex 07, France; 2 Université Lyon1_F-69003, UMR Ruminant mycoplasmoses, VetAgro Sup-Campus Vétérinaire de Lyon, Marcy-L'étoile F-69280, France

## Abstract

**Background:**

Ruminant mycoplasmoses are important diseases worldwide and several are listed by the World Organization for Animal Health to be of major economic significance. In France the distribution of mycoplasmal species isolated from clinical samples collected from diseased animals upon veterinary request, is monitored by a network known as VIGIMYC (for VIGIlance to MYCoplasmoses of ruminants). The veterinary diagnostic laboratories collaborating with VIGIMYC are responsible for isolating the mycoplasmas while identification of the isolates is centralized by the French Food Safety Agency (AFSSA) in Lyon. The VIGIMYC framework can also be used for specific surveys and one example, on the prevalence of *M. bovis *in bovine respiratory diseases, is presented here.

**Results:**

Between 2003 and 2008, 34 laboratories were involved in the network and 1904 mycoplasma isolates, originating from the main ruminant-breeding areas, were identified. For cattle, the high prevalence of *M. bovis *in bronchopneumonia, notably in young animals, was confirmed by VIGIMYC and an associated specific survey, whereas the non-emergence of species such as *M. alkalescens *and *M. canis *was also demonstrated. The etiological agent of bovine contagious pleuropneumonia was never isolated. The principal mycoplasmosis in goats was contagious agalactia with *M. mycoides *subsp. *capri *as main agent. Ovine mycoplasmoses, most of which were associated with pneumonia in lambs, were infrequently reported. One exception was ovine contagious agalactia (due to *M. agalactiae*) that has recently re-emerged in the Pyrénées where it had been endemic for years and was also reported in Corsica, which was previously considered free.

**Conclusions:**

Although VIGIMYC is a passive network and somewhat biased as regards sample collection and processing, it has provided, in this study, an overview of the main mycoplasmoses of ruminants in France. The French epidemiological situation is compared to those existing elsewhere in the world.

## Background

The name "mycoplasma" is conventionally used to designate Mollicutes, a class of bacteria that lack a cell wall and have often been portrayed as "minimal self-replicating organisms" because of their small genome size and the paucity of their metabolic pathways. More than 200 species have been identified to date, amongst which are severe pathogens of humans, animals and plants.

Approximately 40 mycoplasma species have so far been described in domestic ruminants (cattle, sheep and goats). Some of them are pathogenic and 3 mycoplasma-induced diseases are currently listed by the World Organization for Animal Health (OIE) to be of major economic importance. The first is contagious bovine pleuropneumonia (CBPP) of which the causal agent is *Mycoplasma (M.) mycoides *subsp. *mycoides *biotype Small Colony (*Mmm*SC). CBPP was once a major panzootic disease of cattle and is still of consequence in Africa and parts of Asia [1]. Europe has been considered CBPP-free since the beginning of the 20th century despite some localized resurgences [2,3]. The second is contagious caprine pleuropneumonia (CCPP) caused by *M. capricolum *subsp. *capripneumoniae (Mccp)*, a disease that is still rife in Africa and the Middle East [4,5] and has more recently been identified along eastern European borders [6] and in captive wildlife [7]. The third is contagious agalactia (CA) a cosmopolitan disease that affects small ruminants and is a serious threat to dairy production in the Mediterranean basin [8]. The CA syndrome takes many clinical forms (mastitis, arthritis, pneumonia and septicemia) and is caused by several *Mycoplasma *species: *M. agalactiae *affects sheep and goats, while mycoplasmas in the spiroplasma group, consisting of *M. mycoides *subsp. *capri *(*Mmc*) (including the former *M. mycoides *subsp. *mycoides *Large Colony [9]), *M. capricolum *subsp. *capricolum *(*Mcc*) and *M. putrefaciens *mainly affect goats. Another mycoplasmosis in cattle, in which *M. bovis *is the causal agent, is not on the OIE list but is considered to intensify concomitantly with animal production and international trade. It is associated with a variety of clinical expressions [10], notably infectious bronchopneumonia (IBP) in young animals, as previously reported in France [11] and several other countries [12-16].

Increased interest has recently been shown in monitoring mycoplasma incidence and distribution to assess the risks of introduction, emergence, resurgence or intensification of these economically and/or statutorily important diseases. The United Kingdom is the only European country to have set up a national surveillance network and regularly publishes prevalence results ([16], see also the website http://www.defra.gov.uk/vla/reports/rep_surv.htm). The epidemiological status of other European countries can only be inferred from fixed-term surveys (see for instance [12,17,18]). However the coexistence of distinct situations throughout Europe has been documented. For example, the UK is free of all mycoplasmoses listed by the OIE [16] whereas CA is widespread in southern Europe [8].

Since 2003, France has been managing a national epidemiological surveillance network known as VIGIMYC, the aim of which is to determine which mycoplasmas are the usual etiological agents of ruminant diseases. Special emphasis is placed on CBPP, mainly because the persistence of *Mmm*SC in Europe cannot be definitely excluded after the last unexpected and atypical CBPP resurgences that occurred in the 1990's in southwestern Europe, including France [2]. The VIGIMYC operating procedures have been adapted to detect the European form of CBPP, i.e. poorly virulent and immunogenic strains [19,20]. Moreover, because the CBPP agent had been isolated from small ruminants in Africa, Asia and Europe [21-23], these have been considered as potential reservoirs and included in the surveillance strategy.

This article describes the VIGIMYC operating procedures and the principal data obtained between 2003 and 2008. The results of a survey, concerning the relative importance of mycoplasmoses in bovine pneumonia and conducted over a shorter period within the VIGIMYC framework, are also presented. A critical analysis of the derived epidemiological picture is proposed which takes into account the surveillance methodology and data from other countries.

## Methods

### VIGIMYC operating procedures

VIGIMYC is managed by the French Food Safety Agency (AFSSA) in Lyon under the guidance of a steering committee that includes representatives from partner-laboratories, public authorities, veterinarians, farmers and scientists. The day-to-day functioning of this centralized and passive network depends on several protagonists. Whenever the veterinary practitioner in charge of a herd suspects a mycoplasmosis, clinical specimens are sampled and sent to a local laboratory for mycoplasma culture. Information about the clinical case is attached to each sample. If a mycoplasma is isolated, the culture is sent to the AFSSA laboratory for identification of the isolates, which requires special procedures and cannot rely on biochemical characterization, unlike more common bacteria [24]. The results of mycoplasma identification are sent back to the local laboratory and forwarded to the veterinary practitioner. All identification results, together with a description of the clinical cases, are registered in an AFSSA database.

### Survey of incidence of mycoplasmosis in cattle respiratory diseases

In 2008 a questionnaire was sent to all VIGIMYC partner-laboratories to assess the number of clinical specimens from bovine respiratory diseases received per year and the number of such specimens that were seeded for bacteria and/or mycoplasma detection. A total of 23 laboratories answered the questionnaire for the 2006-2007 period and the number of samples specifically positive for *M. bovis *was determined.

### Isolation and identification of isolates

The isolation [25] and culture [24] of mycoplasmas were performed as previously described. Both broth and agar cultures were sent to AFSSA for identification purposes.

The isolates were identified by dot immunoblotting on a filtration membrane [26]. Each isolate was tested on specific hyperimmune sera prepared from reference strains of the 11 most commonly isolated ruminant mycoplasmas and a specific *Mmm*SC monoclonal antibody [27]. In the event of an ambiguous or negative response to all sera, further assays were conducted using species-specific PCR and/or house-keeping genes sequencing, mainly 16S rDNA [28] but also *fus*A for strains belonging to the "*M. mycoides*" group [29,30].

## Results

**Figure 1:**
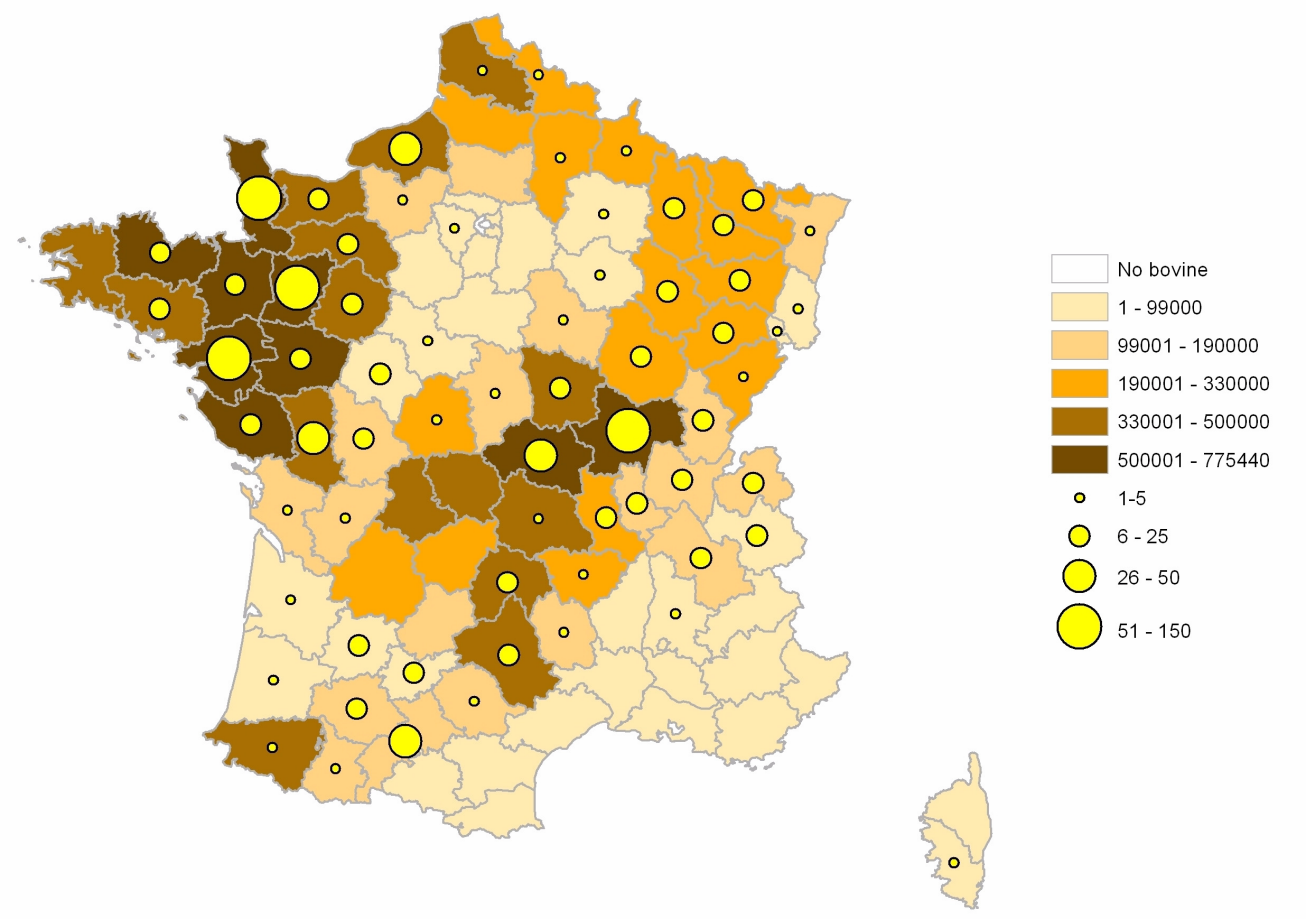
**Distribution of bovine breeding areas in France and origin of mycoplasmal cultures sent to AFSSA for identification**. The color code of each department indicates the number of bovines present in this department in 2008 (Agrest 2008, http://agreste.agriculture.gouv.fr). The yellow circles indicate the number of mycoplasmal cultures originating from each department over the 2003-2008 period. Note that 63 of the 1061 cultures collected are not represented on the map because the indicated origin was ambiguous.

**Figure 2:**
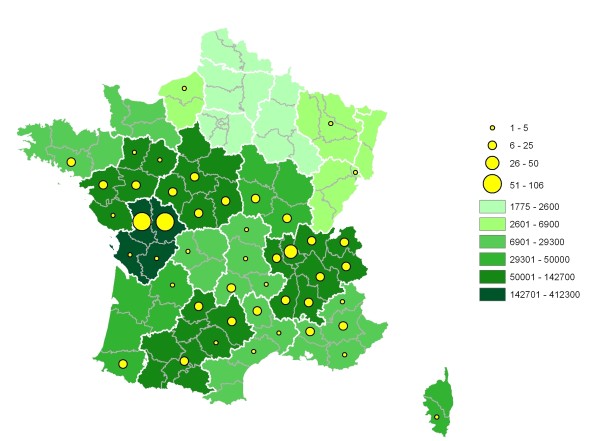
**Distribution of goat-breeding areas in France and origin of mycoplasmal cultures sent to AFSSA for identification**. The color code of each region (a group of departments delineated by a white line) indicates the number of goats present in this region in 2008 (Agrest 2008, http://agreste.agriculture.gouv.fr). The yellow circles indicate the number of mycoplasmal cultures originating from each department over the 2003-2008 period. Note that 96 of the 611 isolates collected are not represented on the map because the indicated origin was ambiguous and one because it originated from an overseas French department.

During the 2003-2008 period, 34 laboratories located in the main ruminant-breeding areas (see Fig. 1 and 2, for cattle and goats, respectively) took part in the network. Clinical specimens were collected from 1312 outbreaks in 64 French departments. A total of 1904 isolates were cultured by local laboratories and sent to AFSSA for identification. These were respectively from cattle (56%), goats (32%), sheep (8%) and wildlife (4%). Most of the 1904 isolates were unambiguously identified by MF-dot immunobinding assays - the technique used for routine mycoplasma identification in the laboratory; see Methods section- and only about forty had to be analyzed further by PCR or sequence analysis to ensure accurate identification.

The overall results are summarized in Table 1. As a general rule 1) the number of mycoplasmal cultures received at AFSSA differs from the number of strains identified, this corresponding to a balance between samples containing several strains and negative samples; 2) each outbreak can be represented by one or several cultures as a function of its importance; and 3) each diseased wildlife animal was considered as an outbreak *per se*.

**Table 1:** Number and distribution of ruminant mycoplasma species, identified by VIGIMYC between 2003 and 2008.

Ruminant hosts (total number of strains)	Cattle^a^ (1142)		Goats^b^ (664)		Sheep^c^ (137)		Ibex^d^ (61)
**Mycoplasma species recovered**	**N**	**%**	**N**	**%**	**N**	**%**	**N**

*M. bovis*	**628**	55	**-**	-	**-**	-	**-**
*M. agalactiae*	**1**	0.1	**13**	2	**28**	20.4	**11**
*M. bovirhinis (o)*	**314**	27.5	**-**	-	**1**	0.7	**-**
*M. canis (p ?)*	**2**	0.2	**-**	-	**-**	-	**-**
*M. arginini (o ?)*	**162**	14.1	**65**	10	**92**	67.1	**4**
*M. alkalescens (p ?)*	**14**	1.2	**-**	-	**-**	-	**-**
*M. canadense (p ?)*	**5**	0.4	**-**	-	**-**	-	**-**
*M. bovigenitalium (o)*	**4**	0.4	**-**	-	**1**	0.7	**-**
*M. mycoides *subsp. *capri*	**4**	0.4	**281**	42.3	**6**	4.4	**43**
*M. mycoides *subsp. *mycoides SC*	**-**	-	**-**	-	**-**	-	**-**
*M. capricolum *subsp. *capricolum*	**-**	-	**175**	26.3	**-**	-	**-**
*M. leachii*	**-**	-	**-**	-	**-**	-	**-**
*M. putrefaciens*	**-**	-	**104**	15.5	**-**	-	**-**
*M. yeatsii (o)*	**-**	-	**24**	3.5	**-**	-	**-**
*M. ovipneumoniae (p ?)*	**-**	-	**1**	0.2	**8**	6	**-**
*M. auris (o)*	**-**	-	**-**	-	**-**	-	**3**
*M. conjunctivae*	**-**	-	**1**	0.2	**-**	-	**-**
*Acholeplasma laidlawii (o)*	**8**	0.7	**-**	-	**1**	0.7	**-**

Species for which the pathogenicity is unknown (p?) or that are opportunists (o) or putative opportunists (o?) are indicated. N = number of strains identified for each species. % = percentage of strains belonging to a mycoplasmal species in relation to the total number of mycoplasmal strains isolated from each farm species. - = zero.

a) Cattle: 1061 cultures were received from 857 outbreaks, of which 61 were negative (no mycoplasma or contamination by bacterial or fungi)

b) Goats: 611 cultures were received from 268 outbreaks, of which 45 were negative.

c) Sheep: 158 cultures were received from 113 outbreaks, of which 28 were negative.

d) Ibex: 74 cultures were received from ibex, of which 13 were negative.

### Cattle

A total of 857 outbreaks in cattle were documented, stemming from 64 departments (Fig. 1), and 1061 cultures were received at AFSSA. Most of these cultures originated from animals with respiratory disease (83%) and from young animals (70%). Mastitis, arthritis or otitis only accounted for 4% of the cultures, while the clinical history was not documented for another 13%. In all, 1142 mycoplasma strains belonging to ten species were identified (see Table 1), the most frequent being *M. bovis *(55%), which was often (12%) mixed with other mycoplasma species, mainly *M. bovirhinis *and *M. arginini*. Seven other species were identified occasionally, including species for which cattle is not the usual host, e.g. *M. canis*, *Mmc *or *M.agalactiae*. The etiological agent of CBPP, *Mmm*SC, was never isolated.

In 2006/2007, a specific survey was conducted within the VIGIMYC framework (for details, see Methods section). This survey revealed that the possible mycoplasmal etiology of bovine respiratory diseases had been frequently investigated in France and confirmed the place of *M. bovis *as a major bovine pathogen. In all 2020 bovine samples were collected from respiratory diseases and analysed in the 23 participating laboratories. Of these, 1557 (75%) were tested for the presence of mycoplasmas, approximately 25% (378/1557) were found positive and 15% (240/1557) specifically contained *M. bovis*.

### Goats

A total of 611 samples from 45 departments (Fig. 2) were received at AFSSA. Most were associated with CA outbreaks (268 documented outbreaks) but some were collected from bulk tank milk controls (25%). In all, 664 strains were identified belonging to eight species (see Table 1), the most frequent being *Mmc *(42%), *Mcc *(26%) and *M. putrefaciens *(15%). *M. agalactiae *was the main etiological agent in only 10 of the 268 CA outbreaks (distributed across 6 departments). *M. arginini *was rarely identified (8% of all strains) and mainly in association with pneumonic pasteurellosis in kids. *M. yeatsii*, a usual commensal of the ear canal, was isolated once from a milk tank and once in a case of septicemia.

### Sheep

A total of 113 outbreaks, mainly of respiratory diseases in lambs, occurred in 36 departments and 158 cultures were received at AFSSA. The most frequently identified mycoplasma was *M. arginini *(67%) while recovery of *Mmc *and *M. ovipneumoniae *was poor. Since 2002, ovine CA due to *M. agalactiae *in France had been only detected in the Pyrénées-Atlantiques department. Historically, this is a major area of endemic ovine CA. However, the department did not take part in VIGIMYC during the 2003-2008 period and therefore most of the CA outbreaks (up to 135 in 2008) were detected by systematic PCR screening of tank milk. Nevertheless, two new CA outbreaks were documented in Corsica in 2008, thanks to VIGIMYC.

### Wildlife

An abnormal increase in mortality, with lesions of severe bronchopneumonia and keratoconjunctivitis, was observed incidentally in certain ibex (*Capra ibex*) populations in the Alps. In addition to common bacterial agents of pneumonia, *M. agalactiae *was regularly isolated from deep lung tissue lesions in these animals. Mycoplasmas from the *Mmc *taxon were also frequently isolated but mainly from the usual body sites of carriage such as the ear canals.

## Discussion

### VIGIMYC operating procedures

This overview of ruminant mycoplasmoses in France over six years cannot be considered exhaustive due to several biases introduced by the VIGIMYC design. Firstly, an investigation for mycoplasmas in a clinical specimen is only carried out by laboratories if explicitly requested by the veterinary practitioners. This request is influenced both by the veterinarian's awareness of mycoplasmoses and by financial considerations. In an attempt to limit these biases AFSSA regularly organizes information sessions about mycoplasmas in ruminants and provides free identification of pre-cultured mycoplasmas. The fact that the detection of mycoplasmas is dependent on a cultivation step introduces another bias since non-cultivable mycoplasmas, such as hemoplasmas [31], or fastidious ones are missed. For example, *M. dispar *is potentially pathogenic for cattle [32] but cannot be grown on the culture media commonly used in laboratories, neither can *Mccp*, the etiological agent of CCPP, a disease currently absent from France. In addition, when several mycoplasma species are present in one sample (as was the case for 16% and 13% of the goat and cattle samples, respectively, analyzed here), the cultivation step tends to favor the most rapidly-growing species to the detriment of the slower growing but often pathogenic ones. Typically, 2 mycoplasma species, *M. bovirhinis *and *M. arginini*, were frequently isolated from cattle and from all ruminants, respectively, but their presence in a sample is of no diagnostic significance since their pathogenicity has never been established [33,34]. However, in sheep, *M. arginini *may mask the existence of a pathogenic slow-growing species like *M. ovipneumoniae*. Last but not least, as explained in detail in a previous study [24], the antigenic identification of mycoplasmas by MF-dot can be ambiguous. Additional identification procedures were notably helpful when mycoplasma species were recovered from unexpected hosts. Recently, the strict host-specificity of several mycoplasmas, such as *Mmm*SC, *M. agalactiae, M. bovis *and *Mcc *[35,36] has been increasingly questioned and notably, in the present study, cattle were found hosting *Mmc *and *M. agalactiae*.

Despite these biases, VIGIMYC provides an informative overview of the epidemiological situation in France for the most commonly found ruminant pathogenic mycoplasmas.

### High prevalence of *M. bovis *infections

The VIGIMYC data and results from the associated survey both confirmed the high prevalence in France of *M. bovis *in IBP as previously demonstrated for young animals [11]. A comparable situation exists in several countries [12-16] although the reported bronchopneumonia infection rates appear to vary considerably with rearing conditions in the different studies. In Europe, Finland, that remains free of *M. bovis*, is a notable exception [17].

However, because *M. bovis *was associated with other pathogenic bacteria in 70% of the clinical cases in the VIGIMYC results (data reported by partner laboratories), its etiological implication has yet to be clarified. *M. bovis *alone was shown to produce lung lesions, under experimental conditions, even in gnotobiotic calves [34] and was seldom isolated from healthy animals in herds [12,37]. Furthermore *M. bovis *appears to initiate the infectious process [11] and causes chronic bronchopneumonia with typical caseous necrosis. Because this infection is persistent and poorly responsive to antibiotics it is undoubtedly one of the most economically deleterious forms of *M. bovis *infection [13,15].

Other clinical forms of *M. bovis *infection such as arthritis, mastitis and otitis, were infrequently reported by VIGIMYC but may be worrisome in other countries [14,38]. Arthritis was often associated with IBP and a few epizooties of arthritis alone in young animals, with unfavorable prognosis, were documented. In addition, the first French case of *M. bovis *otitis in calves was detected in 2008.

### Other mycoplasma infections in cattle

*M. canis *and *M. alkalescens*, suspected to cause emerging epizootic diseases in UK cattle [16,39] were rarely reported by VIGIMYC. *M. canis*, a usual host of the canine genital tract, was isolated from cattle for the first time in 1993 in the Netherlands [40]. By 1999, it represented 12% in Belgium [12] and 18% in the UK [16] of all mycoplasmas isolated from calves with IBP. Its pathogenicity for cattle under experimental conditions is equivocal [41,42]. During the six years covered by this report, *M. canis *was only twice isolated in France. A retrospective study on collection of the AFSSA strains was conducted because of a suspected misidentification between the closely related *M. canis *and *M. bovirhinis *species. This misidentification was confirmed and three *M. canis *strains, respectively isolated in 1985, 1991 and 1995, were re-identified by 16SrDNA sequencing. Consequently, *M. canis *can be considered as present in France since the 80's without causing any epizootic IBP. Likewise, *M. alkalescens *was first isolated in France in 1993 but has seldom been isolated since then and most often in association with *M. bovis*. This situation contrasts with that observed in the UK where the proportion of *M. alkalescens *association with IBP increased from nothing to 16% between 2000 and 2005 [39].

*M. leachii*, the former *M. species *bovine serogroup 7 [9], that was associated with serious epizootic outbreaks of mastitis, arthritis and abortions in Australian cattle [43] and sporadically isolated from bovine and goats in Europe [9,44] was not recovered in France during the 2003-2008 period. Several strains isolated from goats were antigenically similar to PG50, the reference strain of *M. leachii*, but proved however to be genetically closer to *Mcc *or *Mmc *[30]. In addition, no "heterologous-host" reservoir of the CBPP etiological agent was detected by VIGIMYC.

### Contagious agalactia (CA) is still endemic in France

The second major outcome of this study is the confirmation that CA is still widely distributed in France, in contrast to the situation in the UK where CA has never been identified in sheep and goats, despite continuous surveillance [16].

The main etiological agents in goats are mycoplasmas belonging to the *M. mycoides *cluster, and primarily *Mmc*. *Mmc *has previously been described as a cause of latent enzootic infections, with carriage and shedding of multiple strains and sporadic emergence of highly pathogenic and persistent clones [45]. However, strains isolated from clinical and normal samples cannot be differentiated by molecular sub-typing and both have been shown to be pathogenic under experimental conditions (F. Tardy, personal communication). Factors triggering the passage from latent to pathogenic have yet to be identified. Nonetheless, because of this situation the economic impact of caprine CA is probably underestimated in a country that ranks fifth in world goat milk production [46].

Contrary to the situation in mainland Spain [47], *M. agalactiae *is not a major goat pathogen in France and only a few sporadic outbreaks were reported that were widely distributed across the country. In contrast, for sheep, *M. agalactiae *remains problematic in the historically endemic basin of the Western Pyrénées where the disease reoccurred in 2008 with 98 new outbreaks compared with 33 registered in 2006 (J. Vialard, personal communication). All these outbreaks might be due to a single circulating clone that regularly re-emerges (X. Nouvel, personal communication). In contrast, recent introduction of the disease into 2 ovine flocks in Corsica might have resulted from commercial exchange of animals with Sardinia where ovine CA is still endemic [48]. The present VIGIMYC report also shows that *M. agalactiae *might cause pneumonia in the alpine ibex. Fortunately, this enzootic disease occurs only in ibex and no CA has been detected since 2000 in local domestic goats despite the possibility of contacts between goats and ibex in this area.

## Conclusions

Overall, the VIGIMYC data show that several major mycoplasmal diseases of ruminants were documented in France between 2003 and 2008, but that no major evolution in epidemiological trends occurred, and notably no new emergence and no re-emergence of CBPP.

As detailed in a previous study [24], the VIGIMYC surveillance procedures, especially the strain identification step, encounter several difficulties associated with the rapidly evolving nature of mycoplasmas [49]. Recent findings, such as genomic plasticity, putative evolution in quantum leaps, capacity to cross species barriers and intermediary strains, are modifying our current understanding of mycoplasmal diseases, together with their epidemiology and diagnosis. Future surveillance systems will no longer be able to rely on etiological diagnosis that focuses exclusively on currently known pathogenic species. A single generic test able to detect and distinguish any mycoplasmal species from clinical specimens is still being sought.

## Authors' contributions

MC took part in network coordination, results analysis and preparation of the maps in Figs. 1 &2. FT is involved in managing the network, improving its methodology and helped to write the manuscript. DL assisted in the clinical epidemiology follow-up. DC was involved in initiating VIGIMYC and actively promotes its scientific interest to the French regulatory authorities. FP initially conceived the network and drafted the manuscript. All authors read and approved the final manuscript.
